# A robust parameterisation for the analysis of survival data in the presence of covariates with extreme value observations

**DOI:** 10.1186/1745-6215-12-S1-A141

**Published:** 2011-12-13

**Authors:** Richard Jackson, Trevor Cox

**Affiliations:** 1 Liverpool Cancer Trials Unit, Liverpool University, Liverpool, L69 3BX, UK

## 

We propose a new parameterisation for standard survival models which allow for the effect of a covariate with extreme value observations. We show that the robust model can offer an improved fit to both the parametric exponential model and the Cox proportional hazards model. Furthermore, we demonstrate the application of this approach using data from a pancreatic cancer dataset.

Under traditional survival models, covariates enter the model via:

λ=exp{*βx*}

Whilst this allows for ease of interpretation, we show that that this can be a dangerous assumption in the presence of extreme value observations and can lead to misleading survival estimates.

We propose two models which aim to counter this effect. For parametric models, the robust model is of the form:

This model is bounded below at $a/c$ and has an upper asymptote equal to $b$. There are four parameters to be estimated. For the Cox proportional hazards model, the robust model is amended to be:

Here there are three parameters to be estimated. The function equals unity when $\beta= 0$ so that a covariate that is not associated with survival will keep the hazard ratio equal to unity.

These models are fitted to both the parametric exponential and the Cox proportional hazards models in a Frequentist and Bayesian framework using the statistical packages R and WinBUGS. Simulated datasets are used to illustrate the scenarios under which these models are of most use. Initial results show that these models can improve the fit to the data in the presence of extreme value observations when compared using Akaike's Information Criterion (AIC).

Having demonstrated the benefits of robust models, the models are used on a pancreatic dataset. We compare the fit of the post operative CA19-9 value, which has been shown to be a good indicator for overall survival. The robust model here provides a better fit for both the exponential model and the Cox proportional hazards model. We suggest this can lead to improved prognostic models for predicting survival and can also improve comparisons of other effects in a clinical trial by reducing the residual variation observed in the data.

Model residuals are used to assess any departure from the proportionality assumption. These show that extreme value observations are less likely to violate the proportionality assumption when the robust parameterisation is used. An influence function has also been produced to demonstrate the effect of single observations on the model parameters.

